# New Insights into the Pivotal Role of Iron/Heme Metabolism in TLR4/NF-κB Signaling-Mediated Inflammatory Responses in Human Monocytes

**DOI:** 10.3390/cells10102549

**Published:** 2021-09-27

**Authors:** Dong Young Kang, Nipin Sp, Eun Seong Jo, Jin-Moo Lee, Kyoung-Jin Jang

**Affiliations:** 1 Department of Pathology, Institute of Biomedical Science and Technology, School of Medicine, Konkuk University, Chungju 27478, Korea; kdy6459@kku.ac.kr (D.Y.K.); nipinsp@konkuk.ac.kr (N.S.); 2 Pharmacological Research Division, National Institute of Food and Drug Safety Evaluation, Osong Health Technology Administration Complex, Cheongju-si 28159, Korea; eses0706@korea.kr (E.S.J.); elzem@korea.kr (J.-M.L.)

**Keywords:** iron metabolism, heme biosynthesis, LPS, high glucose, inflammatory response, TLR4/NF-κB

## Abstract

Iron metabolism and heme biosynthesis are essential processes in cells during the energy cycle. Alteration in these processes could create an inflammatory condition, which results in tumorigenesis. Studies are conducted on the exact role of iron/heme metabolism in induced inflammatory conditions. This study used lipopolysaccharide (LPS)- or high-glucose-induced inflammation conditions in THP-1 cells to study how iron/heme metabolism participates in inflammatory responses. Here, we used iron and heme assays for measuring total iron and heme. We also used flow cytometry and Western blotting to analyze molecular responses. Our results demonstrated that adding LPS or high-glucose induced iron formation and heme synthesis and elevated the expression levels of proteins responsible for iron metabolism and heme synthesis. We then found that further addition of heme or 5-aminolevulinic acid (ALA) increased heme biosynthesis and promoted inflammatory responses by upregulating TLR4/NF-κB and inflammatory cytokine expressions. We also demonstrated the inhibition of heme synthesis using succinylacetone (SA). Moreover, N-MMP inhibited LPS- or high-glucose-induced inflammatory responses by inhibiting TLR4/NF-κB signaling. Hence, iron/heme metabolism checkpoints could be considered a target for treating inflammatory conditions.

## 1. Introduction

Inflammatory responses occur because of inflammation caused by foreign substances, such as chemicals, bacteria, heat, or toxins. The immune system regulates the inflammatory system through an innate immune response by producing molecular signaling to protect against inflammasomes [1]. As inflammation is considered the primary pathogenic method of chronic diseases, such as diabetes, bowel diseases, or cancer, it needs to be immediately diagnosed before it becomes worse [2]. Hence, knowing the mechanism of signaling pathways that activate inflammatory responses is as essential as their diagnosis.

Inflammatory responses begin by cell surface receptor signaling, where toll-like receptors (TLRs) that are considered key receptor molecules recognize harmful stimuli and send signals to activate inflammatory pathways. These pathways aid in releasing proinflammatory cytokines to recruit inflammatory cells for inflammatory responses [3,4]. Among TLRs, TLR2 and TLR4 play a vital role in the inflammatory response by activating molecular signaling [5]. These TLRs promote signals to the cytoplasm, where many molecules receive the signal and respond. Among them, nuclear factor-kappa B (NF-κB), which is activated, mediates inflammatory and immune responses through the canonical or noncanonical NF-κB pathway [6,7]. The activation of NF-κB leads to its translocation from the cytosol to the nucleus to act as a transcription factor [8] and bind to the promoter of proinflammatory cytokines, such as tumor necrosis factor-α (TNF-α), cyclooxygenase-2 (COX-2), interleukin (IL)-1 receptor (IL-1β), or IL-6 [9,10].

Lipopolysaccharides (LPSs) are a crucial component of the outer membrane of Gram-negative bacteria, and they could act as a stimulator for pathogenic damages. LPS can induce an inflammatory response by activating the innate immune system and regulating pathogen-associated molecular patterns (PAMP) [11]. It also limits the inflammatory response produced by the innate immune system by neutralizing bacterial proinflammatory factors, stimulating TLRs to activate the inflammatory pathway [12,13]. A recent study demonstrated the induction of the inflammatory response in skin fibroblast cells by LPS treatment, significantly reduced by natural sulfur compounds through NF-κB signaling regulation [14].

Hyperglycemic conditions could also lead to inflammation and type 1 and type 2 diabetes, and this prolonged inflammation could result in tumorigenesis [15,16]. High glucose levels have been associated with the pathogenesis of atherosclerosis, which is the major cause of death in patients with type 1 and 2 diabetes [17,18]. Accumulation of diacylglycerol occurs because of high-glucose flux and activates protein kinase C (PKC). PKC is related to inflammation by regulating NF-κB signaling to promote inflammatory response mechanisms [19].

Iron is the key component in hemoglobin that plays a vital role in cellular metabolism by its enzymatic roles in essential cellular functions, such as DNA synthesis and damage response, mediation of mitochondrial functions, and factors that regulate cell proliferation and death [20]. The presence of iron in the body is crucial as it is required for oxygen transport, and owing to this ability, reactive oxygen species (ROS) could be generated through biomolecule oxidation, making iron toxic [21]. Hence, iron homeostasis is crucial in iron metabolism because of its evolving role in inflammation. Ferroportin (FPN) and heme oxygenase 1 (HO-1) take part in iron accumulation in macrophages so that M1 macrophages struggle for iron accumulation. This is because ferritin and M2 macrophages export iron to reduce the amount of iron, thus suppressing the expressions of pro-inflammatory cytokines [22]. Therefore, an alteration in iron metabolism could result in the variation of inflammatory responses, which affects serious diseases, such as cardiovascular diseases or atherosclerosis.

Iron metabolism plays a central role in heme biosynthesis. Heme controls several biological functions, such as signal transduction, oxygen transport, and mitochondrial functions, with the association of different cytochromes, myoglobin, or hemoglobin [23]. Heme is an iron-containing porphyrin molecule, and upon the degradation of heme, iron-induced ferritin, CO, and bilirubin are formed as degradation products [24]. In many pathological situations, such as muscle injury, hemorrhage, hematoma, or ischemia/reperfusion, an excessive amount of heme proteins and free heme were found after hemolysis [25]. Furthermore, studies showed that an increased amount of heme promotes pro-inflammatory cytokine expression, and the subsequent release of heme could lead to renal failure due to local inflammation [26,27]. Hence, understanding the role of iron/heme metabolism in inflammatory response induction is an interesting concept for molecular signaling-based targeted therapy against inflammation.

This study demonstrated the role of iron/heme metabolism in LPS- or high-glucose-induced inflammation conditions and the mechanism by which this metabolism regulates key inflammatory pathways as an inflammatory response to LPS and high glucose.

## 2. Materials and Methods

### 2.1. Antibodies and Cell Culture Reagents

The human monocyte cell line, THP-1, was purchased from the Korean Cell Line Bank (Cancer Research Institute, Seoul National University; Chongno-gu, Seoul, Korea; 40202). Roswell Park Memorial Institute-1640 (RPMI-1640) medium, penicillin-streptomycin solution, and trypsin–EDTA (0.05%) were purchased from Gibco (Thermo Fisher Scientific, Inc., Waltham, MA, USA). LPS (L2630), d-glucose (G8270), D-mannitol (M4125), and fetal bovine serum (FBS) were obtained from Sigma-Aldrich (Merck KGaA, St. Louis, MO). Antibodies specific for IL-6 (sc-57315), TLR4 (sc-293072), FECH (sc-377377), and β-actin (sc-47778) with secondary antibody anti-mouse (sc-516102) were purchased from Santa Cruz Biotechnology (Dallas, TX, USA). Antibodies specific for NF-κB (#8242), IL-1β (#12703), and secondary antibody antirabbit (#7074) were obtained from Cell Signaling Technology (Beverly, MA, USA). Antibodies specific for STEAP3 (ab151566), DMT1 (ab55735), transferrin receptor (ab84036), ALAS1 (ab84962), HO-1 (ab137749), and TNF-α (ab183218) were purchased from Abcam (Cambridge, MA, USA). ABCB10 (C-381841) and FLVCR1 (C-750126) antibodies were obtained from Lifespan Biosciences (Seattle, WA, USA). Ferroportin (NBP1-21502) antibody was purchased from Novus Biologicals (Littleton, CO, USA), and MFRN antibody was purchased from MyBioSource (San Diego, CA, USA). Finally, an antibody specific to ABCB6 (PA1723) was obtained from Boster Bio (Pleasanton, CA, USA). We did not validate the true specificity of antibodies ourselves, and hence we provided the catalogue details of each antibody for reference.

### 2.2. Cell Culture and Treatment

THP-1 cells were used in this study as they are considered as a classical model for induced inflammation studies. THP-1 cells were maintained in endotoxin-free RPMI-1640 (Gibco; 22400-089) supplemented with 50 µM mercaptoethanol, 10% FBS, 100 U/mL penicillin, and 100 µg/mL streptomycin at 37 °C and 5% CO_2_. Cells were cultured (1 × 10^6^ cells/mL) for 72 h (Western blotting) in 10 ng/mL LPS, 10 mM mannitol, 5.5 mM glucose, or 25 mM glucose containing the indicated concentrations of heme, ALA, SA, or N-MMP. Then, they were gently washed twice with phosphate-buffered saline (PBS) for further experiments.

### 2.3. Iron Assay Analysis

Iron estimation was performed using an iron assay kit (MAK025) purchased from Sigma-Aldrich (Merck KGaA, St. Louis, MO, USA). Briefly, cells (2 × 10^6^) were homogenized in an iron assay buffer and collected by centrifugation at 16,000 × g for 10 min at 4 °C. Then, the cells were mixed with an iron assay buffer, added to a 96-well plate along with an iron reducer, and incubated in a horizontal shaker for 30 min at 25 °C. Then, 100 μL of the iron probe was added to each well and incubated for 1 h at 25 °C. Absorbance was measured at 593 nm, and controls were set to 100% for comparison.

### 2.4. FACS Analysis for Ferrous Ion (Fe^2+^)

After cultured cells were washed with prewarmed serum-free no-glucose RPMI 1640 medium, cells were stained in 2 mL of the staining solution containing FerroFarRed (5 µM; GC903-01; GORYO Chemical) and incubated in a CO_2_ incubator at 37 °C for 30–40 min. After staining, cells were washed with 1 mL of prewarmed serum-free no-glucose RPMI 1640 medium and used for fluorescence-activated cell sorting (FACS) analysis.

### 2.5. Western Blotting

Whole-cell lysates were prepared by incubating untreated or LPS, mannitol, 5.5 mM glucose, or 25 mM glucose-treated THP-1 cells on ice with the radioimmunoprecipitation lysis buffer (20–188; EMD Millipore) containing protease and phosphatase inhibitors. Protein concentrations were measured using the Bradford method (Thermo Fisher Scientific, Inc., Waltham, MA, USA). The same amounts of protein (100 μg/well) were resolved with 10–15% SDS-PAGE. Separated proteins were then transferred onto nitrocellulose membranes. The blots were blocked for 1 h with 5% skim milk (BD Biosciences, CA, USA) in a TBS-T buffer (20 mM Tris-HCl (Sigma-Aldrich; Merck KGaA, St. Louis, Missouri), pH 7.6, 137 mM NaCl (Formedium, Norfolk, UK; NAC0_3_), 0.1× Tween 20 (Scientific Sales, Inc., Oak Ridge, TN, USA)). The membranes were then incubated overnight at 4 °C in a shaker with primary antibodies diluted in 5% skim milk. The membranes were then washed with TBS-T and incubated for 1 h at room temperature with HRP-conjugated secondary antibodies. Detection was performed using a Femto Clean Enhanced Chemiluminescence Solution Kit (GenDEPOT; 77449; Katy, TX, USA) and a LAS-4000 imaging device (Fujifilm, Tokyo, Japan).

### 2.6. Heme Quantification

Cultured THP-1 cells (2 × 10^5^) were suspended in 500 μL of saturated oxalic acid in water and heated at 100 °C for 30 min. For accurate assays, only the oxalic acid and unheated cells were used as blanks in order to remove the free PPIX background. Emission peaks at 608 and 662 nm using 400 nm excitation were measured using a fluorescence microplate reader (Hitachi MPF-4).

### 2.7. Statistical Analyses

All experiments were performed at least thrice, and results were expressed as the mean ± standard error of the mean. Statistical analyses were conducted by one-way analysis of variance (ANOVA) or Student’s *t*-test. One-way ANOVA was performed with Tukey’s post hoc test. The analyses were performed using SAS v.9.3 (SAS Institute, Inc., Cary, NC, USA). A *p*-value < 0.05 (*) was considered to indicate a significant difference.

## 3. Results

### 3.1. LPS- or Glucose-Induced Inflammation Elevates Iron Metabolism

**Figure 1:**
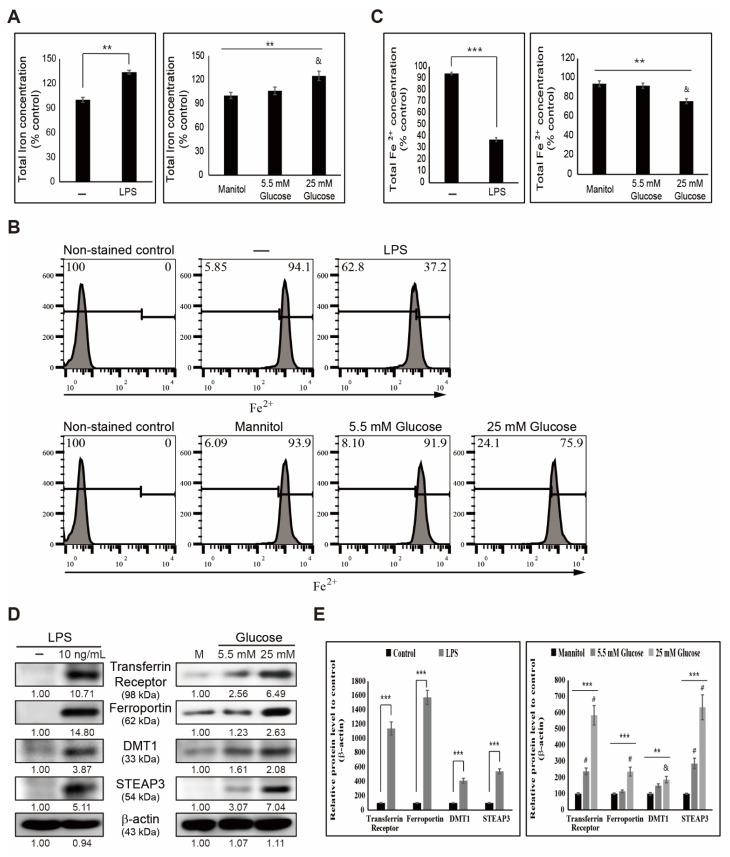
Induced inflammation increases iron metabolism. (**A**) Iron assay showing total iron concentration in THP-1 cells treated with LPS (10 ng/mL) for 48 h and mannitol or glucose (5.5 and 25 mM) for 72 h. Data obtained after three experiments. ** *p* < 0.01, control vs. LPS (*t*-test). ** *p* < 0.01, mannitol vs. high glucose (ANOVA test). The mean difference is significant at the 0.01 level. & *p* < 0.01 vs. control. (**B**) Flow cytometry showing the expression of Fe^2+^ in THP-1 cells after treatment with LPS (10 ng/mL) for 48 h and mannitol or glucose (5.5 and 25 mM) for 72 h. Data are representative of three independent experiments. (**C**) Graphical representation of flow cytometry showing the expression of Fe^2+^ in THP-1 cells after treatment with LPS (10 ng/mL) for 48 h. Data are representative of three independent experiments. *** p < 0.001 (*t*-test). Graphical representation of flow cytometry showing the expression of Fe^2+^ in THP-1 cells after treatment with mannitol or glucose (5.5 and 25 mM) for 72 h. Data are representative of three independent experiments. ** p < 0.01 (ANOVA test). & *p* < 0.01 vs. control. (**D**) Western blotting of transferrin receptor, ferroportin, DMT1, and STEAP3 in THP-1 cells after treatment with LPS (10 ng/mL) for 48 h and mannitol or glucose (5.5 and 25 mM) for 72 h. Expression levels were estimated by densitometry and normalized to β-actin. Data were repeated thrice for confirmation. (**E**) Graphical representation of transferrin receptor, ferroportin, DMT1, and STEAP3 in THP-1 cells after treatment with LPS (10 ng/mL) for 48 h. *** p < 0.001 (*t*-test). Graphical representation of proteins in THP-1 cells after treatment with mannitol or glucose (5.5 and 25 mM) for 72 h. ** p < 0.01. & *p* < 0.01 vs. control. *** p < 0.001 (ANOVA test). # *p* < 0.001 vs. control.

First, we hypothesized that inflammation could contribute to iron metabolism by involving iron production and transport for iron homeostasis. To elucidate the effect of inflammation on iron metabolism, we estimated total iron concentration by adding LPS or high glucose in human THP-1 cells (Figure 1A). Results obtained showed an increase in the amount of total iron with LPS treatment, and a similar increase was also observed in high-glucose conditions (25 mM glucose) compared with mannitol treatment and low-glucose (5.5 mM glucose)-treated cells. This indicated the role of iron in inflammatory conditions. To confirm this, we analyzed the amount of ferrous ion (Fe^2+^) using flow cytometry in THP-1 cells with or without LPS and high glucose (Figure 1B). The results showed suppression in Fe^2+^, indicating an increase in iron transport as Fe^2+^ was converted to ferric ion (Fe^3+^) so that an increased amount of total iron is converted for iron metabolism (Figure 1C). Finally, we used Western blotting for the proteins responsible for iron transport to demonstrate the molecular mechanism behind it. The results suggested an upregulation in the expression pattern of transferrin receptor and ferroportin by LPS. Additionally, high glucose in THP-1 cells suggested the conversion of Fe^2+^ to Fe^3+^ for iron metabolism and elevation in expression levels of divalent metal transporter 1 (DMT1) (Figure 1D). Moreover, the six-transmembrane epithelial antigen of prostate 3 (STEAP3) proteins confirmed the role of iron metabolism in LPS- or high-glucose-induced inflammation (Figure 1E).

### 3.2. LPS- or Glucose-Induced Inflammation Enhances Heme Biosynthesis

**Figure 2:**
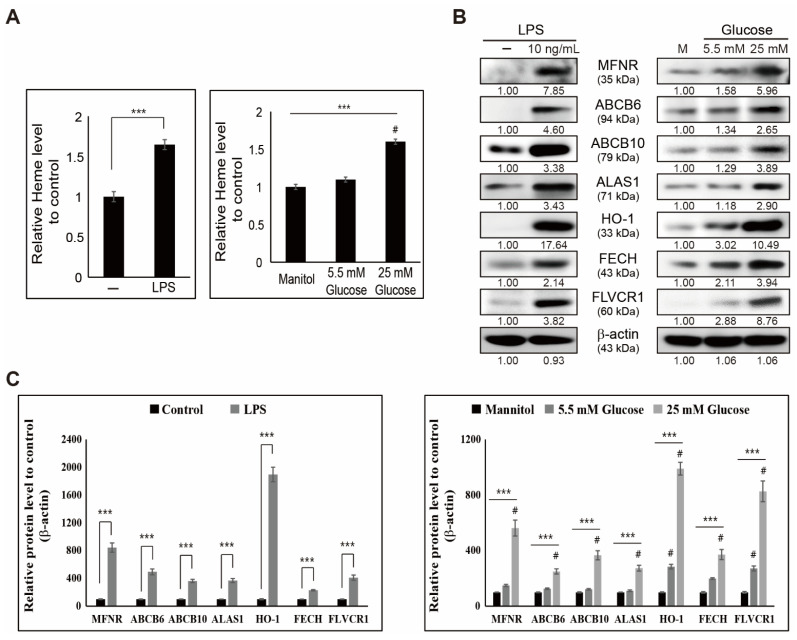
Heme biosynthesis occurs during inflammation. (**A**) Heme assay showing the relative heme level obtained in THP-1 cells after treatment with LPS (10 ng/mL) for 48 h and mannitol or glucose (5.5 and 25 mM) for 72 h. Data obtained after three experiments. *** *p* < 0.001, control vs. LPS (*t*-test). *** *p* < 0.001, mannitol vs. high glucose (ANOVA test). # The mean difference is significant at the 0.001 level. (**B**) Western blotting of MFRN, ABCB6, ABCB10, ALAS1, HO-1, FECH, and FLVCR1 proteins in THP-1 cells after treatment with LPS (10 ng/mL) for 48 h and mannitol or glucose (5.5 and 25 mM) for 72 h. Expression levels were estimated by densitometry and normalized to β-actin. Data were repeated thrice for confirmation. (**C**) Graphical representation of MFRN, ABCB6, ABCB10, ALAS1, HO-1, FECH, and FLVCR1 proteins in THP-1 cells after treatment with LPS (10 ng/mL) for 48 h. *** p < 0.001 (*t*-test). Representative expression of proteins in THP-1 cells after treatment with mannitol or glucose (5.5 and 25 mM) for 72 h. All data were repeated three times for confirmation. *** p < 0.001 (ANOVA test). # *p* < 0.001 vs. control.

We saw an increase in iron metabolism with inflammation, and here, we hypothesized that elevated iron results in heme biosynthesis. Heme biosynthesis was analyzed using a heme assay, and the results obtained showed an increase in heme levels by adding LPS and high glucose in THP-1 cells (Figure 2A). A significant enhancement in heme indicated the possible role of iron/heme metabolism during inflammation. To confirm heme biosynthesis due to inflammation, we analyzed the molecular pattern of proteins responsible for heme production using Western blotting (Figure 2B). The results showed significant upregulated expression patterns of proteins responsible for heme biosynthesis, such as mitoferrin (MFRN), ATP-binding cassette super-family B member 6 (ABCB6), ABCB10, delta-aminolevulinate synthase 1 (ALAS1), HO-1, ferrochelatase (FECH), and feline leukemia virus subgroup C receptor-related protein 1 (FLVCR1), with LPS or high-glucose (Figure 2C) treatment in THP-1 cells. These results indicated that iron/heme metabolism plays a key role in inflammation.

### 3.3. Heme Biosynthesis Induces Inflammatory Responses through TLR4/NF-κB Signaling in THP-1 Cells

**Figure 3:**
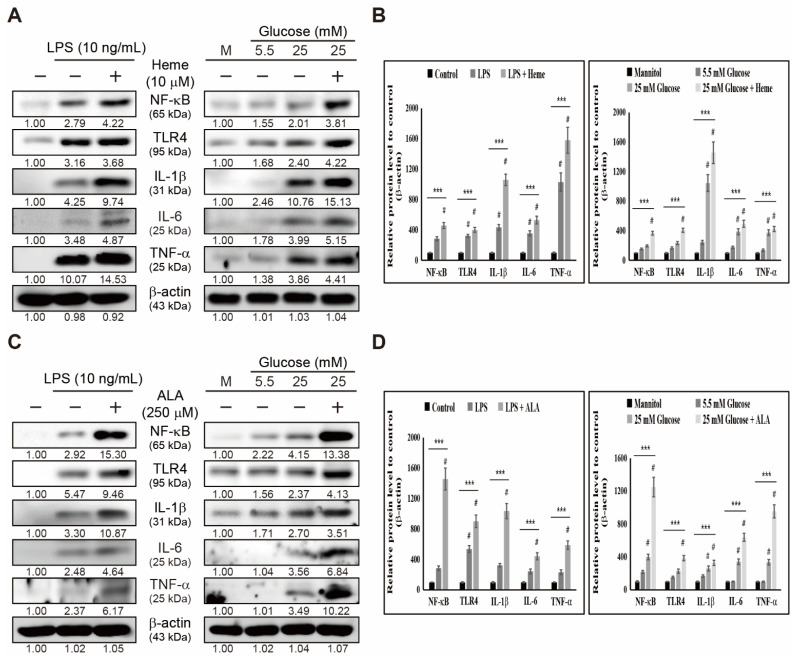
Heme biosynthesis induces inflammatory responses. (**A**) Western blotting of TLR4, NF-κB, IL-6, IL-1β, and TNF-α proteins in THP-1 cells after treatment with heme (10 µM), LPS (10 ng/mL) for 48 h, and mannitol or glucose (5.5 and 25 mM) for 72 h. Expression levels were estimated by densitometry and normalized to β-actin. Data were repeated thrice for confirmation. (**B**) Graphical representation of TLR4, NF-κB, IL-6, IL-1β, and TNF-α proteins in THP-1 cells after treatment with heme (10 µM) and LPS (10 ng/mL) for 48 h. *** p < 0.001 (ANOVA test). # *p* < 0.001 vs. control. Graphical representation of TLR4, NF-κB, IL-6, IL-1β, and TNF-α proteins in THP-1 cells after treatment with heme (10 µM), and mannitol or glucose (5.5 and 25 mM) for 72 h. Data were repeated thrice for confirmation. *** p < 0.001(ANOVA test). # *p* < 0.001 vs. control. (**C**) Western blotting of TLR4, NF-κB, IL-6, IL-1β, and TNF-α proteins in THP-1 cells after treatment with ALA (250 µM), LPS (10 ng/mL) for 48 h, and mannitol or glucose (5.5 and 25 mM) for 72 h. Expression levels were estimated by densitometry and normalized to β-actin. Data are representative of three independent experiments. (**D**) Graphical representation of TLR4, NF-κB, IL-6, IL-1β, and TNF-α proteins in THP-1 cells after treatment with ALA (250 µM) and LPS (10 ng/mL) for 48 h. *** p < 0.001 (ANOVA test). # *p* < 0.001 vs. control. Graphical representation of TLR4, NF-κB, IL-6, IL-1β, and TNF-α proteins in THP-1 cells after treatment with ALA (250 µM), and mannitol or glucose (5.5 and 25 mM) for 72 h. Data were repeated thrice for confirmation. *** p < 0.001 (ANOVA test). # *p* < 0.001 vs. control.

Based on our findings, we observed the role of iron/heme metabolism in inflammation. Here, we analyzed whether these metabolisms could enhance inflammatory responses through the inflammation pathway. First, we treated THP-1 cells with 10 μM heme and LPS or high glucose and then analyzed the proteins by Western blotting (Figure 3A). The results obtained suggested an increase in the expression levels of receptor protein TLR4, key inflammatory response protein NF-κB, and pro-inflammatory cytokines IL-6, IL-1β, and TNF-α upon treatment with LPS or high glucose (Figure 3B). Treatment with heme further significantly elevated the expression of these inflammatory response pathways, suggesting the role of heme biosynthesis in the inflammatory response. To confirm this, we used 5-aminolevulinic acid (ALA) for the treatment in THP-1 cells (Figure 3C), and results showed a similar result as that observed with heme treatment, so that adding LPS or high glucose (Figure 3D) significantly elevated the expression levels of the inflammatory response pathway. Furthermore, ALA further upregulated the expression levels of these proteins. As ALA is considered an intermediate in heme biosynthesis, it is evident for the role of heme biosynthesis in inflammatory responses through TLR4/NF-κB signaling.

### 3.4. Inhibition of Heme Biosynthesis Causes Inflammatory Response Suppression in THP-1 Cells

**Figure 4:**
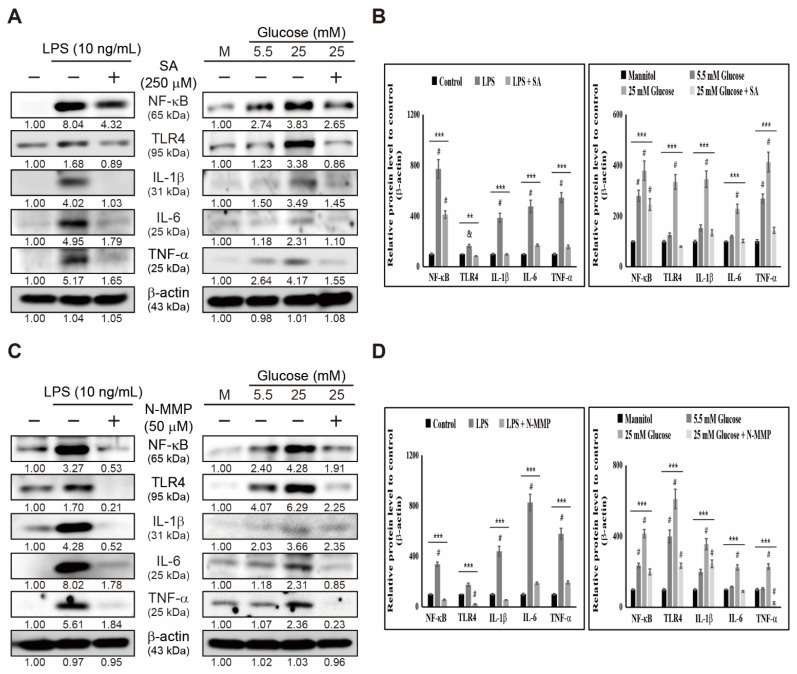
Inhibiting heme synthesis suppresses inflammatory responses. (**A**) Western blotting of TLR4, NF-κB, IL-6, IL-1β, and TNF-α proteins in THP-1 cells after treatment with SA (250 µM), LPS (10 ng/mL) for 48 h, and mannitol or glucose (5.5 and 25 mM) for 72 h. Expression levels were estimated by densitometry and normalized to β-actin. Data are representative of three independent experiments. (**B**) Graphical representation of TLR4, NF-κB, IL-6, IL-1β, and TNF-α proteins in THP-1 cells after treatment with SA (250 µM) and LPS (10 ng/mL) for 48 h. ** p < 0.01. & *p* < 0.01 vs. control. *** p < 0.001 (ANOVA test). # *p* < 0.001 vs. control. Graphical representation of TLR4, NF-κB, IL-6, IL-1β, and TNF-α proteins in THP-1 cells after treatment with SA (250 µM) and mannitol or glucose (5.5 and 25 mM) for 72 h. Data were repeated thrice for confirmation. *** p < 0.001 (ANOVA test). # *p* < 0.001 vs. control. (**C**) Western blotting of TLR4, NF-κB, IL-6, IL-1β, and TNF-α proteins in THP-1 cells after treatment with N-MMP (50 µM), LPS (10 ng/mL) for 48 h, and mannitol or glucose (5.5 and 25 mM) for 72 h. Expression levels were estimated by densitometry and normalized to β-actin. Data were repeated thrice for confirmation. (**D**) Graphical representation of TLR4, NF-κB, IL-6, IL-1β, and TNF-α proteins in THP-1 cells after treatment with N-MMP (50 µM) and LPS (10 ng/mL) for 48 h. *** p < 0.001 (ANOVA test). # *p* < 0.001 vs. control. Graphical representation of TLR4, NF-κB, IL-6, IL-1β, and TNF-α proteins in THP-1 cells after treatment with N-MMP (50 µM) and mannitol or glucose (5.5 and 25 mM) for 72 h. *** p < 0.001 (ANOVA test). # *p* < 0.001 vs. control.

**Figure 5:**
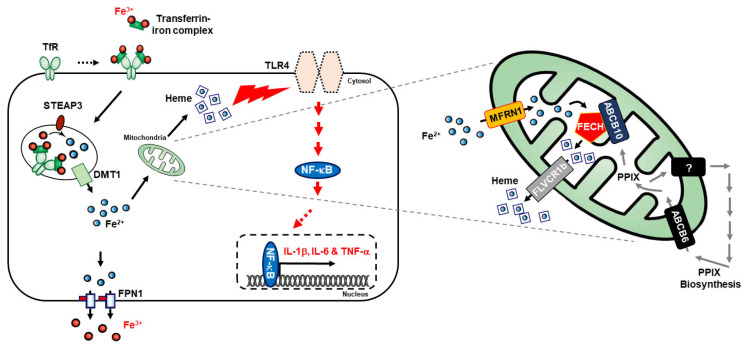
Molecular regulatory mechanism of iron metabolism and heme biosynthesis in inflammatory responses by regulating the TLR4/NF-κB pathway, and thereby mediating pro-inflammatory cytokines, IL-6, IL-1β, and TNF-α.

We observed an elevation in the inflammatory responses by adding heme and its intermediate ALA in THP-1 cells, suggesting the possible role of heme biosynthesis in inflammatory responses. To confirm this, we inhibited heme biosynthesis and then analyzed the expression patterns of the inflammation pathway. First, we treated THP-1 cells with succinylacetone (SA), an inhibitor of heme synthesis, along with LPS and high glucose, and then analyzed the expression levels of the inflammatory response pathway by Western blotting (Figure 4A). The results suggested a significant increase in the expression levels of TLR4, NF-κB, IL-6, IL-1β, and TNF-α protein by LPS or high-glucose (Figure 4B) treatment. In contrast, treatment with SA successfully suppressed those elevated levels. This result suggested the absence of inflammatory responses when heme is inhibited. To confirm this, we treated THP-1 cells with LPS or high glucose and N-methyl mesoporphyrin IX (N-MMP), which could block heme biosynthesis (Figure 4C). The results suggested a similar significant outcome as that observed with SA treatment, as N-MMP suppressed LPS- or high-glucose-induced expression of inflammatory pathway proteins TLR4, NF-κB, IL-6, IL-1β, and TNF-α (Figure 4D). These results demonstrated the central role of heme biosynthesis in the inflammatory response induced by LPS or high glucose in THP-1 cells. The mechanism lying in LPS or high glucose induced inflammation condition depends on iron homeostasis through regulating the iron transport and heme biosynthesis and thereby regulates TLR4/NF-κB signaling to induce inflammatory response through IL-6, IL-1β, and TNF-α (Figure 5).

## 4. Discussion

Inflammation occurs as a response to external stimuli that could negatively affect health. Therefore, knowing the inflammation and inflammatory response mechanism is a better approach for diagnosis and effective treatment. Even though many factors cause inflammatory responses, the molecular signaling mechanism plays a vital role in target-specific therapies. External stimuli, such as LPS or high-glucose conditions, induce inflammation by activating TLRs and sending signals to NF-κB through the canonical or noncanonical NF-κB pathway [28]. Canonical NF-κB activation is possible by activating the IKK complex, leading to inflammatory cytokine activation as a response to inflammation [29]. TLRs also activate NF-κB through PKCα-dependent phosphorylation of ERK1/2 and p38 MAPK, which mediates the release of proinflammatory cytokines [30,31]. Hence, TLR4 and NF-κB regulation decide the ability to induce an inflammatory response to stimuli.

Inflammation and iron metabolism are interconnected because iron is essential to health and iron homeostasis plays a vital role in inflammation [32,33]. We found that LPS- or high-glucose-induced inflammation increased iron concentration, so that the possible iron transport or iron metabolism occurs during inflammation or as a response to inflammation. The reduced amount of inactive ferrous iron (Fe^2+^) in LPS- or high-glucose-treated cells also indicated the role of iron in inflammation through iron transport. To manage iron balance, transferrin receptor 1 (TFR1) and ferroportin 1 (FPN1) regulate homeostasis through iron transport, where TFR1 takes up ferric iron (Fe^3+^) for metabolism and FPN1 is the iron export transmembrane [34]. Our results showed an elevation in the expression levels of TFR1 and FPN1 in LPS- or high-glucose-induced inflammation, and suggested the role of iron metabolism during inflammation. As both transporters play a vital role in iron homeostasis, inflammation could lead to a dysregulated pattern for these transporters. LPS or high-glucose addition enhanced iron metabolism and transport, which in turn induces the expression of TFR1. As a result, the intracellular iron concentration (Figure 1A) increases which in turn disrupts iron homeostasis. In order to maintain the iron homeostasis, the activity of FPN1 increases which starts to pump iron out of the cytoplasm. DMT1 also acts as a membrane transporter of iron from the endosomal system to cytosol and functions in intestinal iron absorption by reducing Fe^3+^ before the translocation of Fe^2+^ [35,36]. STAEP3 also takes part in iron transport by reducing the released Fe^3+^ to Fe^2+^ inside the endosome [37]. Hence, an increase in the expression levels of DMT1 or STEAP3 also explains the role of iron metabolism. Similar to our hypothesis, adding LPS or high glucose showed an upregulation in the expression levels of DMT1 and STEAP3, which suggested that iron metabolism occurs in response to inflammation.

Heme biosynthesis is essential for metabolism, and iron regulates heme synthesis in mitochondria [38]. Heme biosynthesis begins when glycine and succinyl-coA are combined by ALA synthase to form aminolevulinic acid. Hence, the expression of ALAS1 will be significantly increased during heme synthesis. Then, adding ferrous iron to protoporphyrin IX (PPIX) results in heme synthesis catalyzed by the enzyme FECH [39]. The molecular mechanism behind it mainly depends on the activity of FECH and ALAS1 activation, where ALA formation undergoes different transitions and then directs to mitochondria with the help of the putative importer ABCB6. Moreover, inside the mitochondria and FECH, MFRN imports iron from hematopoietic tissues and takes part in the association with FECH and ABCB10 to produce heme [40,41]. Then, the heme produced is transported toward the cytoplasm with the help of FLVCR1, a mitochondrial heme exporter [42]. Our results showed that adding LPS or high glucose induced an elevation in heme synthesis and the expression levels of MFRN, ABCB6, ABCB10, ALAS1, FECH, and FLVCR1 proteins responsible for heme biosynthesis, along with HO-1 protein. Increased expression of HO-1 upon treatment with LPS or high glucose also suggested the role of free heme upon inflammation. These results indicated that an induced inflammation condition is associated with iron metabolism and thereby heme biosynthesis.

The role of iron/heme metabolism is well-known in inflammation. However, its role in the inflammatory response pathway is unknown. Heme or hemin regulates heme biosynthesis by regulating ALA production [43,44], and ALA promotes heme biosynthesis by regulating the ALAS1 enzyme [45]. Moreover, they could regulate the major inflammatory response pathways that consist of signaling from TLRs to NF-κB and the mediation of proinflammatory cytokines. In that case, it may suggest the possible role of heme in inflammatory responses induced by LPS or high glucose. Our results showed that an enhancement in the expression levels of the inflammatory response pathway by LPS or high glucose was further elevated by adding heme or ALA. We recommended that heme biosynthesis induces inflammatory responses, and therefore targeting heme might be considered in treating inflammation.

Inflammatory response induction by heme is a good approach for target-specific therapy against inflammation. If the inhibition of heme biosynthesis suppresses inflammatory responses, those inhibitors could be considered anti-inflammatory candidate agents. Succinyl acetone (SA) is a potent inhibitor of heme by inhibiting ALA dehydratase, a crucial enzyme in heme production [46]. Moreover, N-MMP is considered to block heme synthesis and induce the activity of ALA synthase by inhibiting ferrous iron into PPIX [47]. Adding SA or N-MMP showed an inhibitory effect on the inflammatory responses induced by LPS and high glucose. Thus, blocking heme production causes a successful blockage of induced inflammatory responses. These results suggested that targeting heme is a convenient method to treat inflammation. Therefore, candidate drugs that could inhibit heme biosynthesis might suppress inflammation.

## 5. Conclusions

In this study, we demonstrated the role of iron metabolism and heme biosynthesis in inflammation conditions. LPS- or high-glucose-induced inflammation elevated iron metabolism and heme synthesis. We also evaluated the role of heme synthesis in forming the inflammatory response by regulating the TLR4/NF-κB pathway and inflammatory cytokines. Inhibition of heme also illustrated the suppression of inflammatory responses, suggesting a possible target-specific therapy by focusing on heme biosynthesis.

## Data Availability

The data presented in this study are available upon request from the corresponding author. The data are not publicly available due to personal reasons.
